# The effect of high altitude on ephedrine content and metabolic variations in two species of *Ephedra*

**DOI:** 10.3389/fpls.2023.1236145

**Published:** 2023-10-16

**Authors:** Mengnan Lu, Wenjia He, Ziyan Xu, Yan Lu, M. James C. Crabbe, Ji De

**Affiliations:** ^1^ School of Ecology and environment, Tibet University, Lhasa, Tibet, China; ^2^ Institute of Fisheries Science, Tibet Academy of Agricultural and Animal Husbandry Sciences, Lhasa, Tibet, China; ^3^ School of Pharmacy, Fudan University, Shanghai, China; ^4^ Wolfson College, Oxford University, Oxford, United Kingdom; ^5^ Institute of Biomedical and Environmental Science & Technology, School of Life Sciences, University of Bedfordshire, Luton, United Kingdom; ^6^ School of Life Sciences, Shanxi University, Taiyuan, China

**Keywords:** *Ephedra*, pseudoephedrine, ephedrine, metabolomics, altitude, environment

## Abstract

*Ephedra* is an important plant in Chinese medicine; however, there are few reports on two species of Ephedra which are distributed at high altitudes from 3000 to 5200 meters. We collected a total of 84 individuals representing five *Ephedra gerardiana* and nine *Ephedra saxatilis* populations respectively located from 3158 to 5200 meters altitude, and determined the relative content of 213 metabolites using UHPLC-MS/MS (Ultra-High-Performance Liquid Chromatography-tandem mass spectrometry). 37 Chemical compositions were annotated using the KEGG (Kyoto Encyclopaedia of Genes and Genomes) database. From the top five significant enrichments in metabolic KEGG pathway analysis, we found a total of 166 compounds belonging to phenylpropanoids, 123 flavonoids, 67 metabolites carried by ABC transporters, and 61 in purine metabolism. We identified the top 8 altitude-related compounds in two species. Ephedrine and pseudoephedrine were found to be associated with altitude in both *E. saxatilis* and *E. gerardiana*. To verify which environmental factors influenced the metabolic content, the soil moisture and temperature of each population site were collected, and quantitative analysis of ephedrine and pseudoephedrine was performed using UHPLC-MS (Ultra-High-Performance liquid chromatography-tandem mass spectrometry). After detection, soil moisture ranged from 0.074 to 0.177 mm^3^/mm^3^, and temperature ranged from 9.7°C to 23.9°C. The content of ephedrine ranged from (0.84 ± 0.49)% to (2.01 ± 0.41)% in *E. saxatilis*, which was positively correlated with soil moisture; the content of pseudoephedrine ranged from (0.72 ± 0.45)% to (1.11 ± 0.57)% and was negatively correlated with soil moisture. In contrast to these results, in *E. gerardiana*, the content of ephedrine and pseudoephedrine was negatively correlated with soil moisture. Furthermore, the trends of alkaloid contents in two kinds of *Ephedra* were similar when the temperature was lower than 17°C even if the sum was various. With the increase in soil moisture and temperature, the total alkaloid content of *E. saxatilis* was higher than that of *E. gerardiana*. When the soil moisture was lower, the alkaloid content of the two *Ephedra* species was higher. These results provide useful data for the future separation of new compounds, and for seed homogeneous growth to determine artificial breeding of *Ephedra* located at high altitudes.

## 1 Introduction

*Ephedra* is an important resource in Chinese medicine (Abourashed et al., 2003), used to treat respiratory diseases. The rich medicinal contents of *Ephedra* have become one of the important Chinese medicinal materials for import and export (Hayashi et al., 2019). Almost all *Ephedra* plants contain ephedrine and pseudoephedrine. Pseudoephedrine has antipyretic and analgesic effects. It can be used to relieve the symptoms of a cold. Ephedrine is an adrenergic receptor agonist and is commonly used in subarachnoid anesthesia (Minami et al., 2020). As its central nervous system stimulatory effects, ephedrine is the main substance in the quality control of drugs containing *Ephedra* (Kondo et al., 1999; Minami et al., 2020). Ephedrine and pseudoephedrine are isomers with the same formula C_10_H_15_NO, and they are members of phenethylamine alkaloids (Tian et al., 2022).

About 300 chemical compounds, including alkaloids, volatile oils, flavonoids, polysaccharides, simple phenylpropanins, condensed tannins, organic acids, and sterols have been found in these plants, using traditional separation methods, such as alcohol extraction, water extraction and other methods for extraction, UPLC (High Performance Liquid Chromatography) and other methods for separation (Tian et al., 2022). There are still many components in *Ephedra* that have not been identified, so the identification of *Ephedra* by conventional means is time-consuming and labor-consuming, and the method of metabolomics can effectively identify and shorten the identification time (Hong et al., 2011). Most evaluation studies have been limited to pharmacodynamic chemical compounds of wild *Ephedra* plants (Zhang et al., 2018; Tian et al., 2022), and there are few reports on chemical compositions in two kinds of *Ephedra* distributed at altitudes over 3000 m, namely, *E.saxatilis* and *E. gerardiana*, which are used as Tibetan medicinal plants. We therefore explored the question of whether the metabolite levels of *Ephedra* change under the influence of altitude. It is generally accepted that the extreme environment at high altitudes is an important factor affecting the medicinal substances of plants (Zare-Maivan et al., 2014). Most research focuses on the evolutionary origin of this plant species (Wang and Zheng, 2010; Puebla et al., 2017). Most species of *Ephedra* are erect or sprawling shrubs and grow at a wide range of altitude areas, from near sea level to 5000 m (Thompson, 1912; Thompson, 1918; Carlquist, 1988). Qin et al. (2013) studied the differentiation and phylogeographic history of *Ephedra* plants in southern Tibet. Most of the *Ephedra* species have low intraspecific variation and lack a strong phylogeographic structure, possibly due in part to clonal reproduction and relatively recent origin. *Ephedra* is one of the plants with strong drought and/or cold tolerance in arid and semi-arid areas of Tibet (Wu et al., 2016; González-Juárez et al., 2020), and the coverage rate of *Ephedra* populations in high-altitude areas of Tibet is lower than that in other areas of China due to perennial water shortage and drought (Wu et al., 2016). Therefore, the physiological and biochemical changes of *Ephedra* with changes of in altitude have not been reported. This study aims to explain the response mechanism of *Ephedra* to altitude gradient by metabolic level.

There are a total of 6 species of *Ephedra* found in Tibet, among which *E. saxatilis* is endemic to Tibet, and *E. gerardiana* is widely distributed on the Tibetan plateau over 4000 m altitude (Qin et al., 2013). These two species are typical gymnosperms of high altitudes. Due to extreme environmental stress (Strong ultraviolet light, large temperature difference between day and night, and extreme drought) at the high altitude of the Tibetan plateau, these two species are limited for exploitation and utilization (Hong et al., 2011). With the increase of altitude, environmental factors also affect the plant growth, which also provides ideas for the potential metabolite changes of *Ephedra* at high altitudes with drought and low temperature.

However, the chemical composition of both species and how their contents change with altitude and different environments is still unknown. Since ephedrine and pseudoephedrine are the main effective components, the content of these two substance were chosen to reveal the above problem. It is important to determine whether their contents in *Ephedra* vary with altitude and whether soil moisture and temperature affect the contents. In this study, metabolite and alkaloid contents of 14 different altitude populations of *E. saxatilis* and *E. gerardiana* were analyzed, and the effects of soil moisture and temperature at different altitudes on the chemical composition and alkaloid contents of ephedrine were analyzed. This will provide an important basis for the artificial cultivation of *Ephedra*.

## 2 Experimental procedures

### 2.1 Plant material and environment data collection

**Figure 1:**
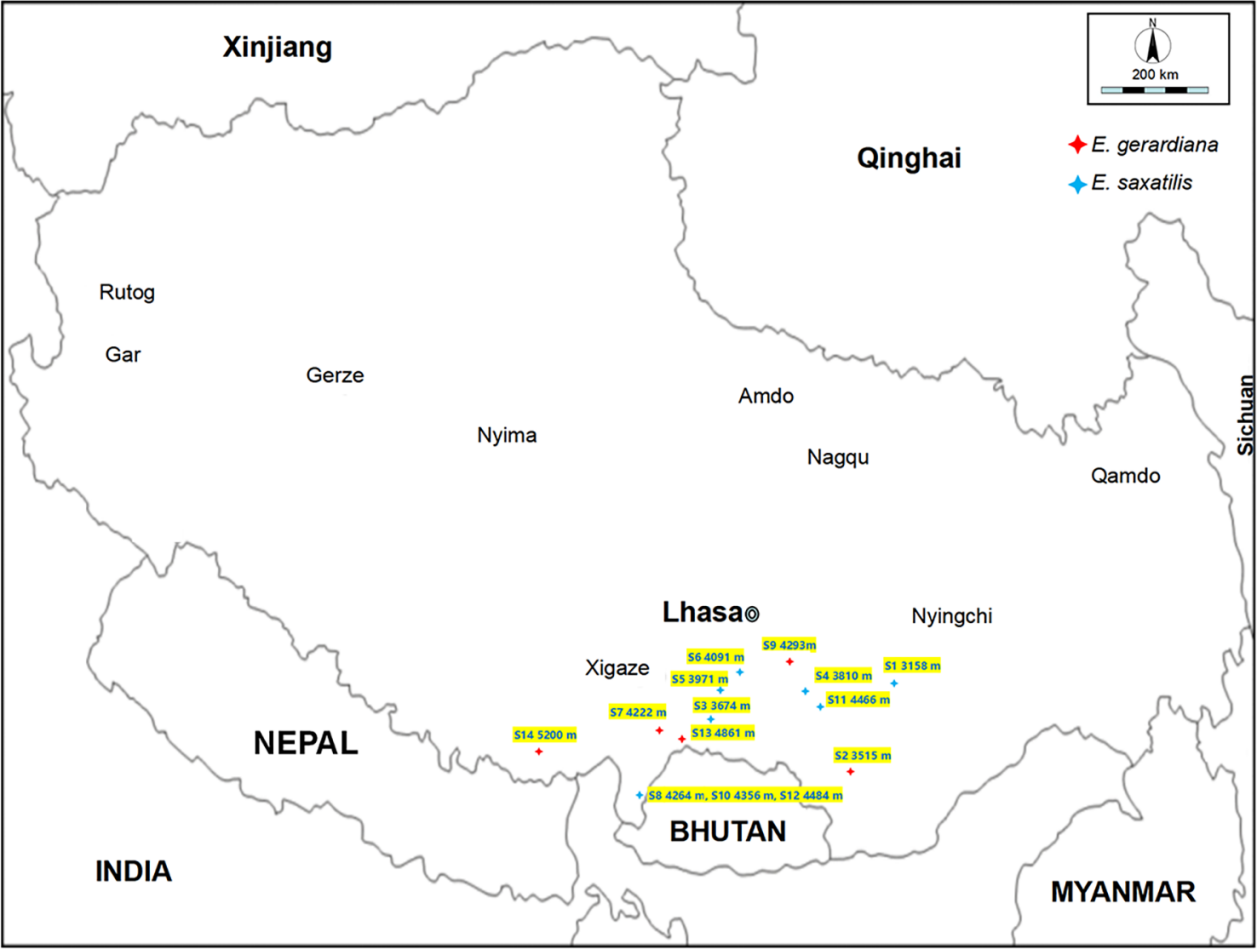
Map of altitude of the sample collection area. *Ephedra* collection site altitude and related information only represents the environmental information at that time.

A total of 84 individuals were collected between June and July 2017 and 2018, representing five *E. gerardiana* populations, and nine *E. saxatilis* populations, respectively located from 3158 to 5200 meters altitude on the Tibet plateau (**Figure 1**; **Table S1**). Six samples were selected from each community, and a total of 84 samples were tested. The species of *Ephedra* were identified by Professor La Qiong, Tibet University. The soil moisture and temperature were measured automatically by inserting a soil measuring thermometer into the soil at 5 cm. The age of the *Ephedra* plants in the growing area was unknown, but the size of the above-ground part of the selected *Ephedra* specimens was the same (about 10-20 cm in diameter and 10-20 cm in height), and the overall shape of the above-ground part was close to the dome. For metabolic profiling, six above-ground plant individuals in each population were collected at the flowering period without regard to plant sex and age. To analyze the correlation between metabolite content and environmental factors, meteorological factors including air temperature, soil moisture, atmospheric pressure, and rainfall (**Table S2**) were collected from World Weather online (https://www.cma.gov.cn/). Soil moisture ranged from 0.074 mm^3^·mm^-3^ to 0.17 mm^3^·mm^-3^ (**Table 1**), and temperatures ranged from 9.7°C to 23.9°C (**Table 1**). The altitude, longitude, and latitude were measured using GPS (Global Positioning System). All *Ephedra* were stored in the refrigerator at -20°C after picking. The samples were freeze-dried after transfer to the laboratory. Freeze-drying condition: (600pa, -20°C).

**Table 1:** Soil moisture and temperature at the sample collection site.

Sample	Soil moisture content/mm^3^·mm^-3^	Temperature/°C
S1	0.162	23.9
S2	0.139	21.7
S3	0.135	20.4
S4	0.143	22.3
S5	0.177	19.6
S6	0.148	19.8
S7	0.101	14.1
S8	0.147	16.4
S9	0.092	15.7
S10	0.116	14.5
S11	0.107	13.2
S12	0.081	11.4
S13	0.087	10.6
S14	0.074	9.7

### 2.2 Metabolite profiling and metabolomics

#### 2.2.1 UHPLC-MS^n^ method

Fifty milligrams of the dried, crushed, stems of *Ephedra* were subjected to 1.0 mL methanol-water (70%, v/v) with adding 20 μL 2-chloro-L-phenylalanine as internal standard, then was ultrasonically extracted for 30 minutes. The liquid was kept at -20°C for 20 min. After centrifuging for 10 min at 14000 × rpm (5000 × g) at 4°C, all *Ephedra* samples were mixed and prepared into QC, preparation conditions, and individual sample conditions, and one QC sample was inserted into every 10 analysis samples during instrumental analysis to examine the repeatability of the entire analysis process. (Blank injection: 1.0 mL methanol-water (70%, v/v) with adding 20 μL 2-chloro-L-phenylalanine as internal standard) (Ren et al., 2022).

200μL extracts were analyzed by LuYan at Fudan University using a Thermo Scientific Dionex Ultimate 3000 UHPLC system with an LTQ Velos Pro Linear Ion Trap Mass Spectrometer (Thermo Fisher Scientific, USA). The analytical conditions were as follows; UHPLC: column, ACE Excel C18 (100 x 3.0mm; Column pore: 3 mm; Particle size: 1.5 μm; Manufacturer: Waters); solvent A, ultra-pure water (Millipore, MA, USA) with 0.1% formic acid, and solvent B, acetonitrile. The gradient program (time(min), B) was as follows, (0.00, 5%); (5.00,10%); (10.00, 10%); (30,25%); (46,55%); (48,100%); (52,100%); (53,5%) and 5min for column equilibration before each injection. The column oven was set to 30°C; the flow rate was 0.3mL min^-1^, and the injection volume was 5μL. The detection wavelength was 254 nm by DAD (Diode Array Detector). One QC sample was injected between 10 samples for testing stability and repeatability.

#### 2.2.2 MS parameters

The mass spectrometer was operated in both ESI positive and negative modes. The linear Ion Trap (LTQ) scan was acquired as a full MS1 scan followed by a data-dependent scan with a mass range of m/z 110–2000. The ESI source operation parameters were as follows: heater temperature, 350°C, capillary voltage, 35V; ion spray Voltage Floating, 3.5 KV; sheath gas flow rate, 40 psi; aux gas flow rate, 10 psi. MRM: Full MS resolution was 70000, and MS/MS resolution was 17500. Data acquisition was performed with the Data Dependent Acquisition (DDA) mode. The detection was carried out over a mass range of 70-1050 m/z. The data collection and analysis were performed using Thermo Xcalibur 2.2 software.

#### 2.2.3 Annotation

After the mass spectrometry detection was completed, qualitative analysis of the metabolite data was analyzed. The raw data of LC/MS was preprocessed for peak picking (Thermo Fisher Scientific™), after date transmission, and alignment using progenesis QI (Thermo) software. The internal standard peaks, as well as any known false positive peaks, including noise, column bleed, and derivatized reagent peaks were removed and the raw signal was smoothed to remove fluctuations. The MS/MS of metabolite peak matching was proposed by the MassFragment™ application manager (Waters Corporation, Milford, USA) and identified using biochemical databases, including KEGG, the Human Metabolome Database (HMDB), and METLIN. The response intensity of the sample mass spectrum peaks was normalized to reduce the errors caused by sample preparation and instrument instability, and the variables of relative standard deviation (RSD)>30% of QC samples were removed using a Majorbio cloud platform (https://cloud.majorbio.com) (Ren et al., 2022).

#### 2.2.4 Preparation of samples and quantitative analysis of ephedrine and pseudoephedrine content annotation

Extraction of ephedrine alkaloids and analysis was performed as described by He et al. (2019) and Zhu et al. (2009) with some modifications. Two hundred grams of *Ephedra* powder was weighed and extracted with 20ml pure methanol containing 1.44% phosphoric acid solution (1:1). After boiling for 2-3 min, it was ultrasonically treated for 30 min. Following centrifugation at 5000 r/min (1800 × g) for 10 min, the supernatant was absorbed and filtered through a microporous membrane (Microporous filter membrane washed with ultra-pure water) with a 0.22 µm pore size for analysis of ephedrine and pseudoephedrine contents using HPLC. The analytical conditions were as follows, HPLC: column, XTERRA® henyl (4.6 mm×250 mm, 5µm); solvent system, methanol (A): 0.092% phosphoric acid aqueous solution added with 0.04% triethylamine and 0.02% dibutylamine (B) (1.5:98.5,v/v); the flow rate was 0.5ml/min-1; column temperature: 30°C; injection volume: 10μl; the detection wavelengths were 210 nm(Chinese pharmacopeia, Ch.P2020 (Chinese pharmacopeia, Ch.P2020). Under these conditions, standard curves were constructed with different concentrations of standards of ephedrine and pseudoephedrine (purchased from https://www.zhzyw.com/).

Note: (total absolute amount of alkaloids/total relative amount of alkaloids) × relative amount parameter = sample alkaloids content.

### 2.3 Statistical analysis

Limma software in the R statistical package (http://www.r-project.org) was used for screening. Differential metabolites among groups were summarized and mapped into their biochemical pathways through metabolic enrichment and pathway analysis based on a database search (KEGG, http://www.genome.jp/kegg/). The data were analyzed through the free online platform of the majorbio cloud platform (cloud.majorbio.com). Ephedrine and pseudoephedrine statistical analyses were performed with SPSS 13.0 (SPSS Inc., Chicago, Illinois, US). The data were analyzed using a two-way analysis of variance (ANOVA) with treatment and time as factors. The means were separated by Duncan’s multiple range test, and differences at P < 0.05 were considered to be significant.

## 3 Results

### 3.1 Metabolic profiling of *Ephedra*, enrichment pathways, and the relationship contents of metabolism with altitude

We determined the relative content of 213 distinct metabolites (**Table S3**) using Ultra High Pressure Liquid Chromatography-tandem mass spectrometry (UHPLC-MS). 37 chemicals were annotated by the KEGG database (**Figure S1A**). 199 metabolites were annotated by the HMDB database, including prenol lipids (22.68%), Glycerophospholipids (20.62%), steroids and steroid derivatives (14.43%), fatty acyls (8.76%), carboxylic acids and derivatives (4.64%) (**Figure S1B**). From the top five significant enrichments of metabolic KEGG pathway analysis, we found a total of 166 compounds belonging to phenylpropanoids, 123 compounds of flavonoids, 67 metabolites carried by ABC transporters, and 61 of purine metabolism (**Figure S2A**).

**Figure 2:**
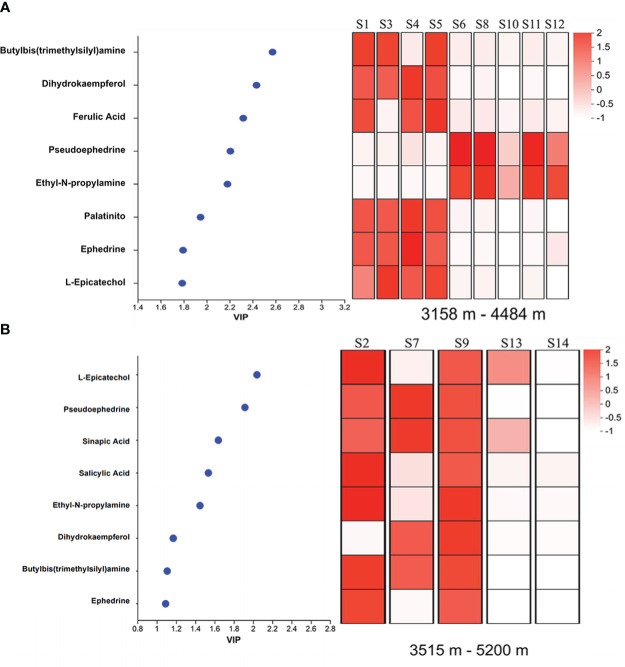
Correlation analyses of metabolites belonging to the phenylpropanoids pathway with altitude. **(A)** shows *E. saxatilis* and **(B)** shows *E. gerardiana*. VIP, Variable Independent Parameters. Each grid in the figure represents the correlation between samples, and each row represents a metabolite. The colors in the figure indicate the relative expression of metabolites in this set of samples. For the specific trend of expression, refer to the number under the color bar at the top right. Different colors indicate the relative magnitude of the correlation coefficient between the samples. The larger the VIP value, the more significant the difference between the samples. The overall hierarchical clustering diagram in the figure normalized the enrichment values and clustered them, with red representing high metabolic enrichment and white representing low metabolic enrichment; Color from red to white, enrichment degree from large to small. The default VIP value > 1 (P < 0.05), and the larger the VIP value, the more significant the direct difference between metabolites.

The relationships between relative content of metabolism and altitude showed that eight compounds in *E. saxatilis*, including butylbis amine, dihydrokaempferol, ferulic acid, pseudoephedrine, ethyl-N-propylamine, ephedrine, palatinitol and L-epicatechol, had significant variations with altitude (**Figure 2A**) and eight compounds in *E.gerardiana*, including L-epicatechol, pseudoephedrine, sinapic acid, salicylic acid, ethyl-N-propylamine, dihydrokaempferol, butylbis amine, and ephedrine, had significant correlation with altitude (**Figure 2B**).

### 3.2 Composition and divination of various ephedrine contents in samples

We used UHPLC for the determination of ephedrine and pseudoephedrine. Correlation analysis was performed according to environmental factors. Among 84 individuals of 14 population samples, the content of ephedrine and pseudoephedrine in the 6 replicated samples varied. The percentage contents of ephedrine, pseudoephedrine, and total alkaloids in each sample community are shown in **Table 2**. The content of ephedrine and pseudoephedrine in *E. saxatilis* ranged from 0.264 mg/mL to 0.042 mg/mL, and from 0.254 mg/mL to 0.017 mg/mL, respectively, and in *E. gerardiana* ranged from 0.137 mg/mL to 0.030 mg/mL, and from 0.265 mg/mL to 0.011 mg/mL, respectively. The total content of these two compounds ranged from 0.336 mg/mL to 0.058 mg/mL in *E. saxatilis*, and *E. gerardiana* ranging from 0.298 mg/mL to 0.041 mg/mL.

**Table 2:** The contents of ephedrine and pseudoephedrine in *E. saxatilis* and *E. gerardiana*.

*E. saxatilis*	*E. gerardiana*	Inter population sample variation	Ephedrine content (mg/mL)	Pseudoephedrine content (mg/mL)	Total(mg/mL)
S1		1	0.178	0.028	0.206
	S2		0.030	0.011	0.041
S3		1	0.264	0.044	0.308
S4		2	0.135	0.068	0.203
S5			0.042	0.017	0.059
S6		2	0.047	0.022	0.069
	S7	1	0.039	0.016	0.055
S8		2	0.067	0.071	0.138
	S9	2	0.044	0.013	0.057
S10		2	0.082	0.234	0.316
S11		4	0.042	0.016	0.058
S12			0.082	0.254	0.336
	S13	1	0.137	0.028	0.165
	S14	3	0.033	0.265	0.298

S1 to S14 represented population samples, values represented mean ± standard deviation with the removal of the intervaried population sample.

### 3.3 Variation of the sum of ephedrine and pseudoephedrine in *E. saxatilis* and *E. gerardiana* with soil temperature and moisture

**Figure 3:**
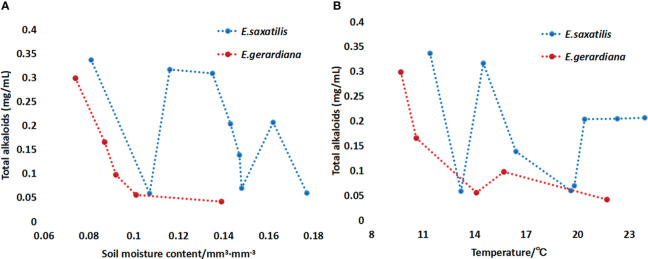
Variation the sum of ephedrine and pseudoephedrine in *E. saxatilis* and *E. gerardiana* with soil mositure **(A)** and temperature **(B)**. Total alkaloid on the vertical is the sum of ephedrine and pseudoephedrine content. The 9 blue dots represent samples collected from 9 populations of *Ephedra* in *E. saxatilis*. The content of ephedrine and pseudoephedrine in 6 individuals from each population was determined, respectively, and the average value is shown by a dot. Similarly, the five red dots represent the sum of ephedrine and pseudoephedrine in the five *E. gerardiana* populations. The vertical line on figure in the histograms indicates the SD.

The metabolome studies showed that the contents of ephedrine and pseudoephedrine in these two species were correlated with altitude. To verify which factors influenced the content of the major compounds of *Ephedra* species, the local climate factors and soil moisture were collected. The correlation of the content of ephedrine and pseudoephedrine with soil moisture was analyzed. The results showed that with an increase in soil moisture and temperature, the sum of ephedrine and pseudoephedrine in *E. saxatilis* first decreased, then increased again, and repeatedly decreased, increased, and finally decreased. In contrast, the contents of *E. gerardiana* decreased with the increase of soil moisture and temperature (**Figure 3**).

### 3.4 Variation trend of ephedrine and pseudoephedrine in *E. saxatilis* and *E. gerardiana* with soil moisture

**Figure 4:**
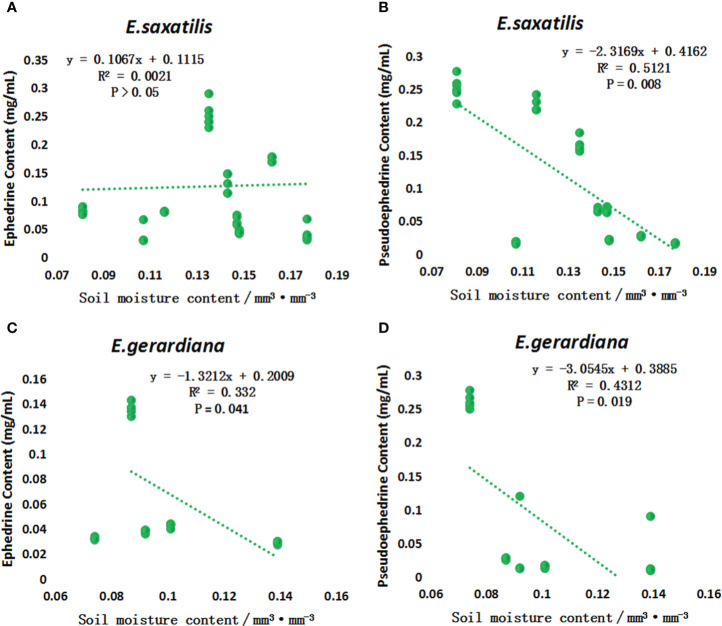
The variation of ephedrine and pseudoephedrine content in *E. saxatilis* and *E. gerardiana* with soil moisture. Variation of ephedrine in *E. saxatilis* with soil moisture **(A)**, Variation of pseudoephedrine in *E. saxatilis* with soil moisture **(B)**. Variation of ephedrine in *E*. *gerardiana* with soil moisture **(C)**, Variation of pseudoephedrine in *E. gerardiana* with soil moisture **(D)**. The value of each point represents the mean value.

The contents of ephedrine and pseudoephedrine were higher in *E. saxatilis* than in *E. gerardiana*. To reveal the influence of soil moisture on the content of these two alkaloids, a correlation analysis was performed. The results showed that the content of ephedrine in *E. saxatilis* was not correlated with soil moisture, while the content of pseudoephedrine was negatively correlated with soil moisture. In contrast to the results above, in *E. gerardiana*, the content of ephedrine was negatively correlated with soil moisture, while the content of pseudoephedrine was also negatively correlated with moisture (**Figure 4**).

### 3.5 Variation trend of ephedrine and pseudoephedrine in *E. saxatilis* and *E. gerardiana* with temperature

**Figure 5:**
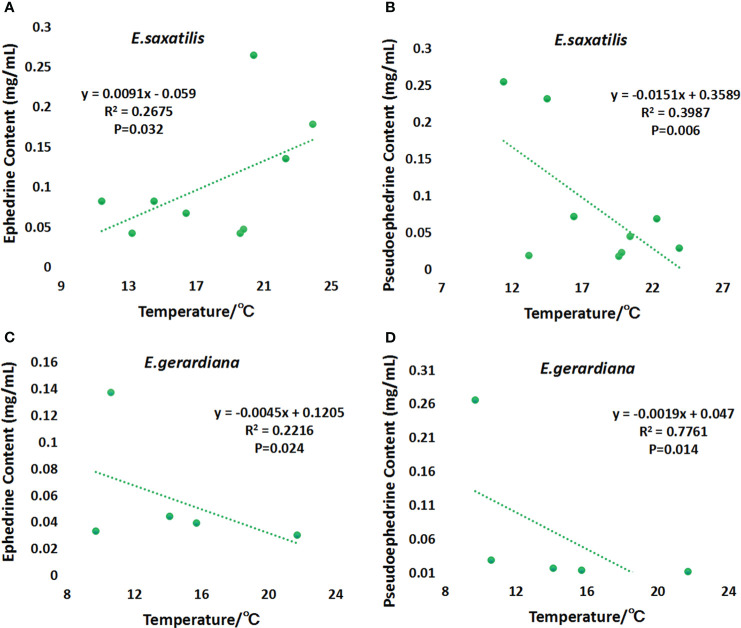
Variation of ephedrine and pseudoephedrine with soil temperature. Variation of ephedrine in *E. saxatilis* with temperature **(A)**, Variation of pseudoephedrine in *E. saxatilis* with temperature **(B)**. Variation of ephedrine in *E. gerardiana* with temperature **(C)**, Variation of pseudoephedrine in *E. gerardiana* with temperature **(D)**. The value of each point represents the mean value.

The correlation of the content of ephedrine and pseudoephedrine with temperature was analyzed. The results showed that ephedrine showed a significant upward trend with temperature in *E. saxatilis* (P < 0.05) (**Figure 5A**). Ephedrine showed a significant decreasing trend with temperature in *E. gerardiana* (P < 0.05) (**Figure 5C**). Pseudoephedrine showed a decreasing trend in both species of ephedrine, the decrease in temperature was significant only in *E. saxatilis* (P < 0.05) (**Figure 5B**). Only the trend relationship in **Figure 5D** is not significant.

## 4 Discussion

### 4.1 Collection environment

The spatial differentiation of the Qinghai-Tibet Plateau ecosystem is mainly determined by the high altitude (Xie et al., 2019). Temperature and precipitation decrease in the northwest, with a warm and humid climate in the southeast and a cold and dry climate in the northwest (Chang, 1981; Chang, 1983). The diverse environmental states at different altitudes lead to different characteristics in vegetation ecosystems. Even for plants in different locations in the same region, there are differences of soil moisture and temperature (Minami et al., 2021). In addition, Tibet’s water reserves are also affected by global warming, and water resources are becoming more abundant (Chu et al., 2018; Fu et al., 2019). The shifts in temperature and soil moisture cause the same plant to produce divergent metabolism (Xiao et al., 2014). Considering the species of *E. saxatilis* and *E. gerardiana* distributed at altitudes from 3,200 m to 5,200 m, the habitat in which they grow varies from humid to arid. Therefore, it can be inferred that their metabolites, including ephedrine and pseudoephedrine, will change with altitude. To reveal which metabolites of *Ephedra* are associated with altitude, the samples were collected from different altitudes, and analyzed the correlation metabolism with soil moisture and temperature.

### 4.2 Ephedrine alkaloid in relation to soil temperature and moisture

In this study, 213 compounds with annotated information were found. However, due to the limitations of the metabolome methods, there are still many compounds that have been reported in the literature, that were not detected in this study. Another reason for these undetected compounds may due to that the plant of *E. saxatilis* and *E. gerardiana* in this study is quite different from other *Ephedra* species that have been reported. There are still many compounds that have not been annotated, which will be our next research direction. Different temperatures can lead to changes in plant genes, as well as changes in plant metabolites. Some plants’ adaptation to temperature is regulated by internal metabolites. For example, 5-Hydroxybuspirone tends to increase with increasing temperature (Ito et al., 2022). Drastic temperature fluctuations caused by climate change adversely affect plant growth and threaten crop productivity. Uncovering metabolites in the plant immune system that are themselves protected from temperature stress is not only a key fundamental question, but also crucial for agricultural sustainability and food security. Ergosterol peroxide can enhance the drought-resistant ability of plants (Ding and Yang, 2022). Ito et al. (2022) found that poor fruit set and growth were caused by temperature stress in tomato cultivation. Low-temperature stress in spring is an abiotic stress limiting the growth and productivity of winter wheat (Li et al., 2022). The photosynthetic characteristics and tolerance of peony decreased at high temperatures, damaging the photosynthetic capacity of peony leaves and their photosynthetic mechanism (Ji et al., 2022). As shown in **Figure 3** of this study, with the increase in soil temperature, the sum of ephedrine and pseudoephedrine content fluctuated in *E. saxatilis*, such as an initial decrease, and then an increase, followed by a decrease again, while in *E. gerardiana* it decreased with soil temperature. This means that the influence of soil temperature on the total ephedrine content for these two kinds of *Ephedra* was different. As shown in **Figure 5**, in *E. saxatilis*, the relative content of ephedrine increased with soil temperature increasing(*s* (P < 0.05) (**Figure 5A**), while the content of pseudoephedrine decreased (P < 0.05) (**Figure 5B**). The relative content of ephedrine and pseudoephedrine deceased with soil temperature (**Figures 5C, D**). From this result, we suggest that the increase of temperature accelerates the synthesis of ephedrine, while the synthesis of pseudoephedrine decreases in *E. saxatilis.* However, it was not fit for *E. gerardiana* to synthesize these two alkaloids in response to higher soil temperature at high altitudes. It would be ideal to increase the sample size to explain this conclusion for *E. gerardiana.*

The above research provides us with ideas to enhance ephedrine content. In the further cultivation of large quantities of *Ephedra*, it would be important to regulate the temperature to ensure the stable content of ephedrine.

Under the stress of drought, high temperature, low temperature, salt and alkali, and toxic pollutants, important substances in plants will accumulate a large amount in cells, which makes these organisms show amazing adaptability to harsh environments (Stracke et al., 2020). Soil moisture is the main factor in plant growth, vegetation restoration, and plant ecology (Sheffer et al., 2020). As shown in **Figure 3**, soil moisture influenced the ephedrine and pseudoephedrine content; the content of ephedrine in *E. saxatilis* increased while the content of pseudoephedrine decreased. In this study, the results showed that with increasing soil moisture and temperature, the sum of ephedrine and pseudoephedrine content fluctuated in *E. saxatilis*, while in *E. gerardiana* it decreased with soil temperature. In contrast, the contents in *E. gerardiana* decreased with an increase in soil moisture and temperature. Similar results have been reported on *E. gerardiana*, Minami et al. (2022). This provides a reference for the changes of alkaloids of the two *Ephedra* species in this study. Changes of chemical compositions in plants with soil moisture have also been found in other plants, but the trends were different, such as the contents of two alkaloids, galanthamine and lycorine of *Lycoris aurea* (*L. aurea*) were increased due to drought stress (Quan and Liang, 2017; Liang et al., 2020). Plant growth and metabolism are affected by temperature, where the differences among species play an important role. Different plants produce different metabolites in response to soil temperature. For example, in carrots, the higher the temperature, the lower the sucrose level (Abreha et al., 2021). The mechanism for variations of metabolisms due to the genotype and the genetic variations of the plant, such as potato, was stressed by water deficit at different altitudes. Metabolic change was due to morphology, and physiological change under drought stress, such as reported in *Chickpea* (Gökmen and Ceyhan, 2015) and *Sorghum* (Abreha et al., 2021). According to this research and reported in other plants, we demonstrated that the content of alkaloid changes in *Ephedra* possibly caused by changes in environmental factors, such as soil moisture and temperature. It can be found that the different species of *Ephedra* response to altitude was different, which may be because of their adaptation metabolism different.

Ephedrine and pseudoephedrine have been the main substances used for the quality identification standards for *Ephedra*. Other compounds belonging to phenylpropanoid pathways, such as palatinitol, Ethyl-N-propylamine, Butylbis (trimethylsilyl) amine, etc., were identified in this research, many of which have been effective in relaxing bronchial smooth muscle, and inhibit diplococcus pneumoniae (Li et al., 2012). There were also many other pharmacoactive substances in *Ephedra*, such as catechin, epicatechin, catechol, tetramethylpyrazine, etc., that have been reported (Tian et al., 2022). However, there are still many difficulties in determining their efficiency, because it needs to isolate individual substances for pharmacodynamic evaluation. Our analyzed compounds provide ideas for screening pharmacodynamic substances and discovering new compounds.

### 4.3 Comparison of ephedrine content in different *Ephedra* species and quality evaluation

The variation of ephedrine and pseudoephedrine content in *Ephedra* growing in different environments is very important for the selection of suitable soil characteristics. (Minami et al., 2022). According to the Chinese Pharmacopoeia (ChP, 2020), the daily dose of *Ephedra* is 2-10 g. The total amount of ephedrine hydrochloride and pseudoephedrine hydrochloride in ephedrine should not be less than 0.8% in terms of alkaloid composition. Hong et al. (2011) systematically compared the content of ephedrine hydrochloride in *E. equisetin* (34 samples collected from 5 provinces in China), *E. intermedia* (23 samples collected from 4 provinces in China), and *E. inica* (7 samples collected from 1 province in China). It was found that the ephedrine alkaloid in *E. equisetin* was between 0.32% and 2.34%, so the quality of 76% of samples of this species meets the standard of the ChP, 2020. Furthermore, 78% of the samples of *E. inica* (the content of ephedrine alkaloid:0.41%-3.40%), and 100% of the samples of *E. intermedia* (the content of ephedrine alkaloid:1.43%-2.97%) met the quality standard of the ChP, 2020. It was reported that the total alkaloid content of *E. gerardiana* collected from three areas in Tibet met the quality standard of the Japanese Pharmacopoeia XVII (JPXVII) (Minami et al. (2022). Whereas, the quality standard for the content in the ChP (2020) is 0.1% higher than in JPXVII. In this research, the content of alkaloids in *E. saxatilis* was between 1.86% and 2.73%, including ephedrine and pseudoephedrine content ranged from 0.84% to 1.87%, and from 0.7% to1.1%, respectively. Similar to its results, the content of alkaloid in *E. gerardiana* was between 1.75% and 2.08%, including ephedrine and pseudoephedrine content ranging from 1.14%-to 1.84%, and from 0.24% to 0.61%, respectively. Besides, the content of ephedrine and pseudoephedrine in *E. saxatilis* and *E. gerardiana* also met the quality standard of the ChP, 2020. In comparison the content of ephedrine, there are little variations between *Ephedra* species, although without any statistical analysis. However, high altitude with water deficit may be more beneficial for *Ephedra* to produce more ephedrine.

The collected samples in this research were mainly distributed in southern Tibet, where exist differences in plant distribution, environment, and plant age. Therefore, we should further research by seeds for homogeneous growth to determine the content of *Ephedra* under different temperatures and moisture stress.

## 5 Conclusion

*Ephedra* is widely distributed in Tibet, and is used for Tibetan medicine, such as “Wu Wei Gan Lu Yu”. However, its shoots are particularly short, which is not conducive to the collection and development of medicinal materials. *Ephedra saxatilis* and *E. gerardiana* are the main species of *Ephedra* distributed gradient with altitudes above 3200 meters, in Tibet. Therefore, we selected these two species to determine the metabolism under variations in soil temperature and moisture. From the result, by correlation analysis for environmental factors with ephedrine and pseudoephedrine alkaloids, we found that the content of ephedrine alkaloids varied with temperature and soil moisture change. With the increase in soil moisture and temperature, the total alkaloid content of *E. saxatilis* was higher than that of *E. gerardiana.*

The ephedrine content in *E. saxatilis* ranged from 0.84% to 2.01%, the pseudoephedrine content ranged from 0.72%-1.11%, and the total alkaloid content ranged from 1.86% to 2.73%, while the soil moisture ranged from 0.081mm^3^/mm^3^ to 0.177mm^3^/mm^3^, and soil temperature ranged from 11.4°C to 23.9°C. In the same way, the ephedrine content in *E. gerardiana* ranged from 1.14% to 1.84%, pseudoephedrine content ranged from 0.24 mm^3^/mm^3^ to 0.61 mm^3^/mm^3^, and total alkaloid content ranged from 1.75% to 2.08%, when the soil moisture ranged from 0.074 mm^3^/mm^3^ to 0.139 mm^3^/mm^3^, temperature ranged from 9.1°C to 21.7°C. Nevertheless, soil moisture and soil temperature affect the content ephedrine and pseudoephedrine variously. With the increase in soil moisture and temperature, the total alkaloid content of *E. saxatilis* was higher than that of *E. gerardiana*. When the soil moisture was lower, the alkaloid content of the two *Ephedra* species was higher. In conclusion, high altitude with water deficit may be more beneficial for *Ephedra* to produce more alkaloids.

## Data availability statement

The original contributions presented in the study are included in the article/**Supplementary Material**. Further inquiries can be directed to the corresponding author.

## Author contributions

ML and JD designed this experiment and wrote this paper. WH collected sample and data. YL and ZX participated in data analysis. MC participated writing this paper. All authors contributed to the article and approved the submitted version.
