# Supplementary material for: The effect of high altitude on ephedrine content and metabolic variations in two species of Ephedra
Source: Front Plant Sci. 2023 Oct 16;14:1236145. doi: 10.3389/fpls.2023.1236145 (PMC10613977; doi:10.3389/fpls.2023.1236145)
Supplement: Supplementary file 1 [file DataSheet_1.docx]

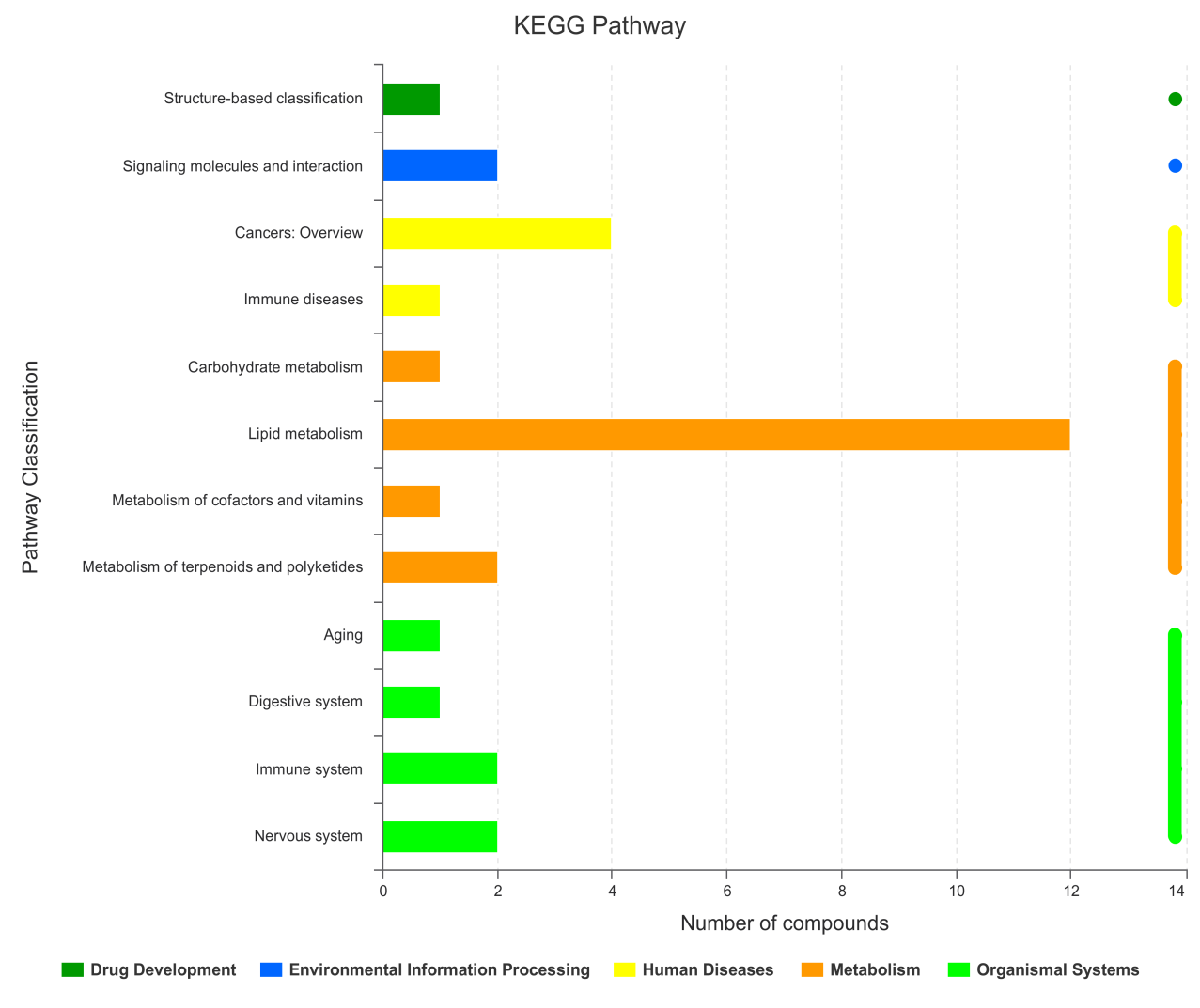


Fig. S1A KEGG Pathway diagram of *Ephedra*

Note: Ordinate is the name of KEGG metabolic pathway and abscissa is the number of compounds annotated to the pathway. The colors of the bars indicate different metabolic pathway categories.


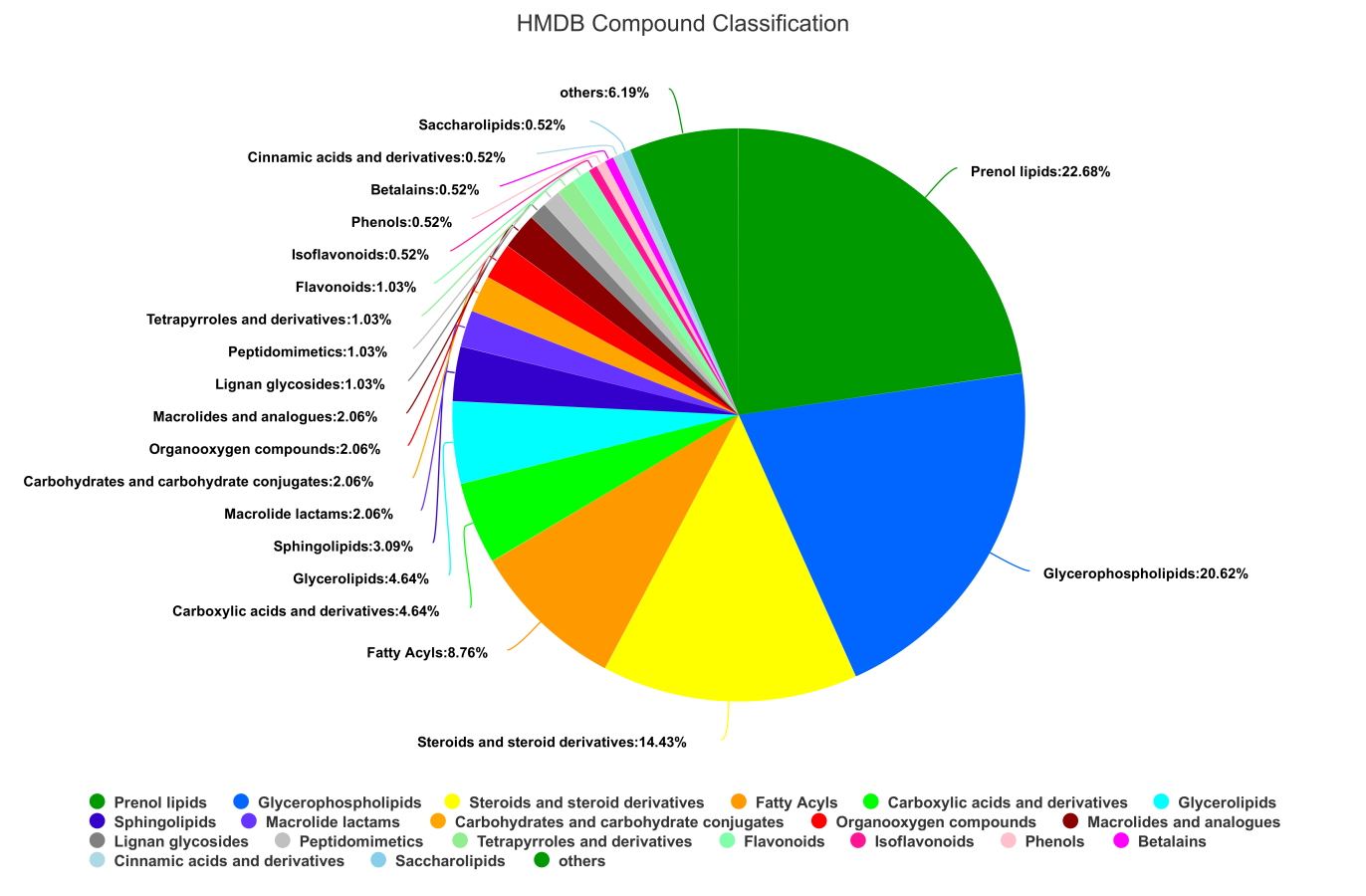


Fig. S1B Metabolite classification map of HMDB class-level layers

Note: The different colors in each pie chart in the figure represent different HMDB classes, and the area represents the relative proportion of metabolites in that class.

Fig. S2A KEGG Pathway sequencing diagram of *Ephedra*

Note: From left to right, the number of metabolites included was ranked from high to low. The higher the column, the more metabolites involved in this pathway in the identified metabolites, and the more active the biological pathway.
